# Modeling suggests that gene circuit architecture controls phenotypic variability in a bacterial persistence network

**DOI:** 10.1186/1752-0509-6-47

**Published:** 2012-05-20

**Authors:** Rachel S Koh, Mary J Dunlop

**Affiliations:** 1 University of Vermont, 33 Colchester Ave, Burlington, VT, 05405, USA

**Keywords:** Persister, Toxin-antitoxin, Gene regulatory network, Feedback

## Abstract

**Background:**

Bacterial persistence is a non-inherited bet-hedging mechanism where a subpopulation of cells enters a dormant state, allowing those cells to survive environmental stress such as treatment with antibiotics. Persister cells are not mutants; they are formed by natural stochastic variation in gene expression. Understanding how regulatory architecture influences the level of phenotypic variability can help us explain how the frequency of persistence events can be tuned.

**Results:**

We present a model of the regulatory network controlling the HipBA toxin-antitoxin system from *Escherichia coli*. Using a biologically realistic model we first determine that the persistence phenotype is not the result of bistability within the network. Next, we develop a stochastic model and show that cells can enter persistence due to random fluctuations in transcription, translation, degradation, and complex formation. We then examine alternative gene circuit architectures for controlling *hipBA* expression and show that networks with more noise (more persisters) and less noise (fewer persisters) are straightforward to achieve. Thus, we propose that the gene circuit architecture can be used to tune the frequency of persistence, a trait that can be selected for by evolution.

**Conclusions:**

We develop deterministic and stochastic models describing how the regulation of toxin and antitoxin expression influences phenotypic variation within a population. Persistence events are the result of stochastic fluctuations in toxin levels that cross a threshold, and their frequency is controlled by the regulatory topology governing gene expression.

## Background

Gene expression is controlled by regulatory networks that influence the mean levels, dynamics, and noise distributions of proteins expressed within a single cell. The outputs of these networks are under selective pressure; thus a regulatory architecture that results in beneficial traits can be selected for by evolution. A key question in systems biology is how the architecture of a gene regulatory network influences the dynamics of gene expression. This question has been explored extensively using mathematical modeling [1]. However, a subtler question is how the architecture of a gene circuit influences the variability in gene expression, and what the implications are for population fitness. Previous studies have shown that similar gene circuit architectures can produce vastly different noise profiles [2,3]. It is clear from systems-level studies that noise in gene networks can be controlled, or selected for, by evolution [4-7]. For example, stress-response genes in *Saccharomyces cerevisiae* have been shown to exhibit significant stochastic variation [8]; similar results have been found for genes that respond to environmental changes [9]. Furthermore, genes that are lethal when deleted exhibit much lower than average levels of stochastic variation [10]. The regulatory architecture of a network also plays an important role in controlling noise [1-4,11-15]. This has been shown specifically for drug-induced stress through several studies demonstrating that increased phenotypic variability can provide a selective advantage [16-19]. The regulation of noise can have dramatic implications when it controls physiological processes important for stress response and survival.

Here, we explore the role of network architecture on the noise properties of a regulatory circuit controlling bacterial persistence. Persistence is a non-inherited mechanism by which bacteria tolerate environmental stress, such as treatment by antibiotics. Cells are able to stochastically switch between a dormant state known as persistence and a regular growth state. In the persistence state, cell growth slows drastically and the cell is therefore immune to treatment by antimicrobial agents that target growth. Examples include beta-lactams, which interfere with cell wall biosynthesis and aminoglycosides, which interrupt translation [20]. Because the cells are in a dormant state, they can effectively evade the drugs. When the cells switch back to the regular growth state, they resume replication and are no longer tolerant to antimicrobial agents. It is important to understand that persistence is not a genetic change; both persisters and normal cells have identical genetic code. Instead, persistence is a transient state that a small subset of the population enters due to phenotypic variation. By maintaining subpopulations of normal and persister cells, the whole population hedges against unlikely but catastrophic events, while still maintaining near optimal growth at the population level.

Persistence plays an important role in chronic infections. High-persistence (or *hip*) mutants are found in *Pseudomonas aeruginosa, Candida albicans, Escherichia coli,* and *Mycobacterium tuberculosis*[21,22]. At any given time, a typical bacterial population will have between 10^-7^ and 10^-5^ cells in the persistence state [23]. High persistence mutants result in 10^-4^ to 10^-2^ portion of cells in the persistence state [20]. This suggests that although it is possible to increase the number of persisters through mutations, their level has been tuned to balance tolerance to environmental threats with a maximal growth rate.

Toxin-antitoxin modules play a key role in the formation of persister cells. Previous work has shown that toxins can induce the dormant state by inhibiting important cellular processes, most commonly mRNA translation [24]. Though toxins have historically been associated with programmed cell death, recent studies show that toxin-antitoxin loci can function to moderate global levels of translation and replication in cells that must survive in stressful environments [21]. For example, the toxins *relE* and *mazF* cleave mRNA, thus inhibiting translation and cell replication. This helps the cell cope with nutritional stress; if there is a temporary shortage of nutrients, some of the population will survive. The action of the toxin can be countered by the presence of a small antitoxin molecule which binds to the large toxin protein, disabling its function [25]. The toxin is stable relative to the antitoxin, whose rapid degradation and production ensure a fast dynamic response. This creates a two-state system, where an excess of toxin will induce dormancy and persistence, but enough antitoxin keeps the cell in its normal growth state. Many pathogenic bacteria have multiple toxin-antitoxin loci. For example, *M. tuberculosis* has 88 known toxin-antitoxin systems [26]. There may be interactions between these different toxin-antitoxin loci that further complicate our current understanding of persistence.

The regulatory architecture of toxin-antitoxin systems is highly conserved across bacterial species [27]. In most cases, the antitoxin gene precedes the toxin gene, with the two loci expressed on the same operon. Because of this, the two genes share a regulatory structure, which can serve to correlate noise in toxin and antitoxin expression. In addition, the antitoxin protein is often a transcription factor, which autorepresses the promoter that controls expression of the two genes. This negative feedback-based inhibition results in low levels of protein expression, which increases the potential for noise in the system. Without feedback, higher toxin and antitoxin expression would mitigate the effect of noise. This tradeoff in noise, which is reduced due to coupled transcription, but elevated due to negative feedback, is the focus of the present study.

The HipBA toxin-antitoxin system from *E. coli* is a well-known persistence mechanism. The HipA toxin causes cell stasis, but HipB can inactivate HipA and create a non-toxic complex. A HipB dimer binds to two copies of HipA, rendering it neutral through conformational inactivation and sequestration [27]. Here, we use the HipBA toxin-antitoxin system as a model for understanding the dynamics and regulatory processes that govern bacterial persistence. HipA is highly conserved among Gram-negative bacteria [27], suggesting that phenotypic variation in toxin-antitoxin expression is a common mechanism for persistence.

The aim of this study is to identify the specific gene expression dynamics that govern persistence. In particular, we ask how the regulatory architecture of the gene circuit leads to noise, and whether this noise is subject to evolutionary tuning. Knowledge of how, why, and when cells switch to persistence can help guide studies on treatment strategies to reduce or eliminate the number of cells that enter persistence.

## Methods

**Figure 1:**
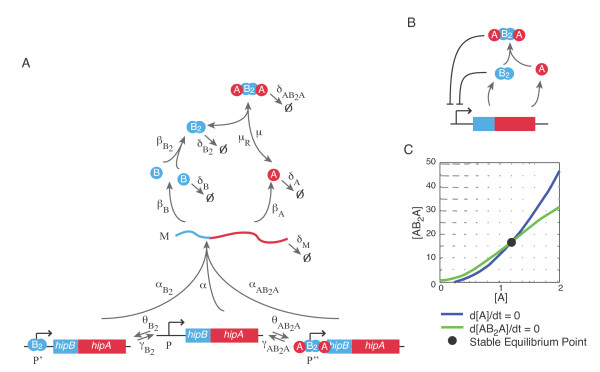
**(A) Biochemical network for the HipBA system.** A is the HipA protein; B is the HipB protein. Dimerized HipB is denoted B_2_, while the complex of HipA and HipB is AB_2_A. P, P’, and P” are the promoter states and M is mRNA. All chemical reactions for the simulations are given in Eq. 2; parameters are listed in Table 1. **(B)** Summary of the dual negative feedback structure of the model. **(C)** Reduced order deterministic model has a single stable equilibrium point. Nullclines for the reduced order model are plotted.

We developed a model of the HipBA toxin-antitoxin system native to *E. coli.* First, we created a deterministic model of the system shown in Figure 1a by using the Law of Mass Action to derive the system in Eq. 1 with parameters given in Table 1. The resulting system describes the temporal dynamics of HipB and HipA expression. The model considers the two genes, *hipB* and *hipA*, which are expressed from the same operon. They are transcribed into mRNA and subsequently translated into proteins. The HipB protein is both transcribed and degraded at a faster rate than HipA because antitoxin proteins are relatively unstable, with a lifetime of a few minutes [21]. HipB dimerizes and then binds to two copies of HipA, which sandwich the dimer [27]. This complex and the HipB dimer can both bind to the promoter site to repress mRNA transcription. Our model specifically focuses on type II persisters, which are generated when cells enter the persistence state stochastically from stationary phase [28]. Differential equations were simulated in Matlab (Mathworks, Inc.) using the ode15s function and analyzed using custom scripts.

(1) $$\begin{array}{c} \frac{d[P]}{dt}=\gamma_{B_{2}}[P^{\prime }]+\gamma_{AB_{2}A}[P^{\prime \prime }]-\theta_{B_{2}}[P][B_{2}]-\theta_{AB_{2}A}[P][AB_{2}A]\\ \frac{d[P^{\prime }]}{dt}=\theta_{B_{2}}[P][B_{2}]-\gamma_{B_{2}}[P^{\prime }]\\ \frac{d[P^{\prime \prime }]}{dt}=\theta_{AB_{2}A}[P][AB_{2}A]-\gamma_{AB_{2}A}[P^{\prime \prime }]\\ \frac{d[M]}{dt}=\alpha [P]+\alpha_{B_{2}}[P^{\prime }]+\alpha_{AB_{2}A}[P^{\prime \prime }]-\delta_{M}[M]\\ \frac{d[B]}{dt}=\beta_{B}[M]-\beta_{B_{2}}{\left[B\right]}^{2}-\delta_{B}[B]\\ \frac{d[A]}{dt}=\beta_{A}[M]-\mu {\left[A\right]}^{2}[B_{2}]+\mu_{R}[AB_{2}A]-\delta_{A}[A]\\ \frac{d[B_{2}]}{dt}=\frac{1}{2}\beta_{B_{2}}{\left[B\right]}^{2}-\frac{1}{2}\mu {\left[A\right]}^{2}[B_{2}]+\frac{1}{2}\mu_{R}[AB_{2}A]-\theta_{B_{2}}[P][B_{2}]+\gamma_{B_{2}}[P^{\prime }]-\delta_{B_{2}}[B_{2}]\\ \frac{d[AB_{2}A]}{dt}=\frac{1}{2}\mu {\left[A\right]}^{2}[B_{2}]-\frac{1}{2}\mu_{R}[AB_{2}A]-\theta_{AB_{2}A}[P][AB_{2}A]+\gamma_{AB_{2}A}[P^{\prime \prime }]-\delta_{AB_{2}A}[AB_{2}A] \end{array}$$

**Table 1:** Chemical reaction parameters for the HipBA system

**Reaction**	**Reactants**	**Products**	**Reaction Rate**	**Value**	**Units**	**Range Tested**	**Reference**
transcription	P	M + P	α	60	hour^-1	6-600	[29,30]
transcription	P'	M + P'	α_B2_	6	hour^-1	0.6-60	[29,30]
transcription	P''	M + P''	α_AB2A_	6	hour^-1	0.6-60	[29,30]
HipB binds to promoter	P + B_2_	P'	θ_B2_	1500	hour^-1/mol	150-15,000	approximated based on [31]
HipB unbinds	P'	P + B_2_	γ_B2_	60	hour^-1	6-600	approximated based on [31]
HipB-HipA complex binds to promoter	P + AB_2_A	P''	θ_AB2A_	1500	hour^-1/mol	150-15,000	[31]
HipBA unbinds	P''	P + AB_2_A	γ_AB2A_	60	hour^-1	6-600	[31]
mRNA degradation	M	0	δ_M_	6	hour^-1	0.6-60	[32]
HipB translation	M	M + B	β_B_	60	mol/hr/mRNA	6-600	[29,30]
HipA translation	M	M + A	β_A_	12	mol/hr/mRNA	1.2-120	[29,30]
HipB degradation	B	0	δ_B_	18	hour^-1	1.8-180	[21]
HipA degradation	A	0	δ_A_	1.2	hour^-1	0.12-12	[31,32]
HipB dimerization	B + B	B_2_	β_B2_	60	hour^-1	6-600	assumed monomeric form to be uncommon
HipB dimer degradation	B_2_	0	δ_B2_	5	hour^-1	0.5-50	[21]
HipB-HipA complex association	B_2_ + 2A	AB_2_A	μ	60	hour^-1/mol	6-600	approximated based on [31]
HipB-HipA complex dissociation	AB_2_A	B_2_ + 2A	μ_R_	60	hour^-1	6-600	approximated based on [31]
HipB-HipA complex degradation	AB_2_A	0	δ_AB2A_	1.2	hour^-1	0.12-12	[31,32]

A represents the HipA protein; B is HipB. B_2_ is the dimerized form of HipB. AB_2_A is the HipA-HipB toxin-antitoxin complex. M is the mRNA transcript from *hipBA*. P is the promoter of *hipBA* with no proteins bound, P’ has B_2_ bound, and P” has AB_2_A bound. The equations for the rate of change of P, P’, and P” describe how the promoter switches between states with nothing, B_2_, and AB_2_A bound. mRNA is transcribed from all three promoter states at different rates and is also degraded. HipA is translated from mRNA and degraded by a protease. HipB is also translated from mRNA and subsequently binds to a second copy of itself to form the HipB dimer, which can bind to and repress the promoter. Dimerization between two HipB molecules is modeled as irreversible because unbinding is slow relative to the binding rate [27,29]. Two copies of HipA bind to one copy of the HipB dimer to form the HipB-HipA complex, which can bind to and represses the promoter.

To analyze the possibility of multiple steady state solutions, we developed a reduced order model and conducted phase plane analysis. The dynamics of the promoter states, mRNA, B, and B_2_ are fast relative to A and AB_2_A. Thus, we assumed that the other states were at equilibrium, and therefore time derivatives equal to zero. The steady state concentrations of the fast variables were used in a two-dimensional model that describes the rate of change of A and AB_2_A. We next used a phase portrait to plot the nullclines, which are the lines where d[A]/dt = 0 and d[AB_2_A]/dt = 0. The points where the nullclines cross are the equilibrium points of the system. If the lines cross more than once, multiple equilibrium solutions are possible. The full equations for the reduced order model are given in the Additional file 1.

Next, we conducted two parametric studies to verify that the system dynamics are monostable for a broad range of biologically realistic parameter values. First, we varied single parameters and checked for the existence of multiple stable states. For each of the parameters in the model we chose that parameter from a log normal distribution using the range given in Table 1. All other parameters were held constant. Using this set of parameters, the system was simulated for 500 distinct initial conditions, which were generated randomly with initial mRNA concentration varying uniformly between 0 and 100 and all proteins varying uniformly between 0 and 1000. Promoter concentrations were held constant for all simulations. We then checked, numerically, whether starting the system at different initial conditions generated any solutions that were more than 1% away from any others. This test was repeated 100 times for each of the 17 model parameters. We found no solutions that varied more than 1% for different initial conditions, suggesting that only monostable solutions exist.

Next, we allowed all system parameters to vary simultaneously. Specifically, all parameters were selected from log normal distributions using the ranges given in Table 1. Initial conditions were selected as described in the single parameter study. We tested 1000 different combinations of parameters and checked for the existence of multiple stable states using the 1% metric described above. For all parameter combinations the systems converged to a single equilibrium point.

Stochastic simulations were performed using Gillespie’s algorithm [33] using custom written C code with subsequent analysis in Matlab. The simulations were based on the chemical reactions shown in Eq. 2 with the parameters listed in Table 1. The simulations were run to generate 1000 hours of data in order to sample the variation of states the system can attain. The model begins by setting the initial numbers of molecules in the system and reaction rates, and then computes the probability of switching to other states based on the chemical reactions and rates. Random variables are used to generate noise in the model and determine when and which reaction occurs based on probability distributions set by substrate levels. The model iterates until the final simulation time is reached. Thus, individual runs show variable levels of each chemical state. In order to avoid biases due to initial transients, we considered only data after the system reached steady state.

(2) $$\begin{array}{l} P\overset{\alpha }{\to }M+P\\ P^{\prime }\overset{\alpha_{B_{2}}}{\to }M+P^{\prime }\\ P^{\prime \prime }\overset{\alpha_{AB_{2}A}}{\to }M+P^{\prime \prime }\\ P+B_{2}\overset{\theta_{B_{2}}}{\to }P^{\prime }\\ P^{\prime }\overset{\gamma_{B_{2}}}{\to }P+B_{2}\\ P+AB_{2}A\overset{\theta_{AB_{2}A}}{\to }P^{\prime \prime }\\ P^{\prime \prime }\overset{\gamma_{AB_{2}A}}{\to }P+AB_{2}A\\ M\overset{\delta_{M}}{\to }0\\ M\overset{\beta_{B}}{\to }M+B\\ M\overset{\beta_{B}}{\to }M+A\\ B\overset{\delta_{B}}{\to }0\\ A\overset{\delta A}{\to }0\\ B+B\overset{\beta_{B_{2}}}{\to }B_{2}\\ B_{2}\overset{\delta_{B_{2}}}{\to }0\\ 2A+B_{2}\overset{\mu }{\to }AB_{2}A\\ AB_{2}A\overset{\mu_{R}}{\to }2A+B_{2}\\ AB_{2}A\overset{\delta_{AB_{2}A}}{\to }0 \end{array}$$

Cells are defined as entering persistence when the number of free HipA toxins exceeds the number of free HipB antitoxins. We used the ratio of free HipA molecules in the cell divided by the sum of free HipA and free HipB molecules to quantify entry into persistence. Hereafter, we will refer to this quantity as R. When R exceeds 0.5 the cell is a persister; below this value the cell is in the normal growth state. This threshold-based approach is consistent with experimental findings from [31], where the levels of HipA and HipB were controlled independently using a plasmid expression system and were found to depend on the concentration of HipB.

The “uncoupled transcription” model replaces the original three promoter states P, P’, and P” with six promoter states, three for *hipB* and three for *hipA*. In addition, the mRNA transcripts M_A_ and M_B_ are now two separate states in the model. Thus, the new model has 12 state variables, but uses the same reaction constants as the native system for the purpose of a controlled comparison (Additional file 1).

The “no feedback” model removes repression of the *hipBA* promoter by B_2_ and AB_2_A. To model this we considered only the P promoter state, eliminating the P’ and P” states from the model. All other reactions and reaction rates are the same as in the original system (Additional file 1).

## Results and discussion

In order to analyze the dynamics of persister formation in the HipBA system we developed a mathematical model based on the regulatory architecture known to control HipB and HipA expression. We first asked how the dynamics of the system led to the formation of persister cells. Next, we studied alternative network architectures to quantify how entry into persistence depends upon gene regulatory structure. In order to address these questions, we developed a biologically realistic model. The system explicitly models promoter states, the binding and unbinding of transcription factors, transcription, translation, complex formation, and degradation. In contrast to previous models [25,31], we consider dimerization, complex formation, multiple modes of repression, and active degradation of the toxin and antitoxins; these processes are modeled based on the physiological findings from experimental studies (Methods).

We first asked how the HipBA regulatory architecture achieves distinct subpopulations of persister and normal cells. A potential mechanism for generating two populations within a group of cells is bistability. There is experimental evidence that isogenic populations can generate bimodal distributions to allow for phenotypic diversity [34,35]. This strategy is beneficial when only a subset of the cells needs to express a particular mechanism, but those cells need to be fully committed to their fate. Positive feedback is known to generate bistable states and can arise from a double negative feedback loop [1]. Therefore, a potential function of the HipBA regulatory network could be to generate two stable states through the use of two negative feedback loops, which act in combination as a positive feedback loop (Figure 1b). In principle, repression of the promoter by the HipB dimer or HipB-HipA complex could lead to a build up of the HipA toxin, and consequently persistence, because the half-life of HipA exceeds that of HipB [21,36]. Alternatively, the higher translation rate of HipB could lead to an excess of antitoxins, leading to the normal growth state. Stochastic fluctuations in gene expression could cause the system to switch between these two states. A previous study proposed a model for persistence based on high cooperativity in a Hill function as a mechanism that generates bistable dynamics [25].

Using our detailed mechanistic model of the biochemical reactions governing HipB and HipA expression we found that the system was monostable for biologically realistic parameter ranges, therefore bistability is not the source of co-existing persister and normal cells. To check for bistable dynamics, we first used time scale separation to develop a reduced order model (Methods, Additional file 1). The dynamics of HipA (A) and the HipB-HipA complex (AB_2_A) were slow relative to the other states in the system. Thus, we developed a reduced order model that assumed other chemical reactants were at steady state relative to A and AB_2_A. We then plotted the nullclines for A and AB_2_A on a phase portrait and showed that, for realistic parameter ranges, they intersect only once (Figure 1c). This single intersection point indicates that only one equilibrium solution exists, thus the system is not bistable.

In order to rule out the possibility that the absence of bistability was the result of the specific parameters used in the model, we conducted two parametric studies (Methods). First, we varied single parameters within a biologically realistic range (Table 1) and tested for bistable behavior over a broad range of initial conditions. In all cases, the solutions converged to a single monostable equilibrium point. Next, we allowed all system parameters to vary at once, and simulated many possible combinations of parameters. Again, solutions for all parameters converged to a single stable point. Through a combination of reduced order system analysis and parametric studies, we find no evidence of bistability in our model of the HipBA system.

An alternative mechanism by which cells can enter persistence is through stochastic fluctuations in gene expression. Random noise in the expression of HipB and HipA can generate phenotypic variability within the population. By chance, some cells within the population will have an excess of the toxin relative to the antitoxin and will enter persistence. To explore the role of phenotypic variability in persister formation, we developed a stochastic model based on the chemical reactions used in the deterministic model. The probabilistic nature of this model more accurately represents the natural fluctuations in the HipBA system.

In order for the HipA toxin to be effective, an individual cell would have to have an excess of free HipA toxins relative to the number of free HipB antitoxins. Thus, the ratio of free HipA molecules to the total number of free HipA and HipB molecules, which we define as R, sets a threshold for persistence. When R exceeds 0.5 a cell has an excess of toxin and can enter persistence. Recent experimental findings suggest that a threshold-based mechanism for persistence, as opposed to bistability, is an accurate representation of the biological origins of persistence [31]. The authors showed that the time spent in the persistence state was proportional to the concentration of excess HipA. Our study examines the entry into persistence, however the duration of the growth arrest period is not calculated by the model, as the dynamics are only valid for non-persister cells. Therefore, our model can be used to simulate the distributions of HipA and HipB and this information can be used to calculate entry into persistence, but not the duration of the growth arrest state.

**Figure 2:**
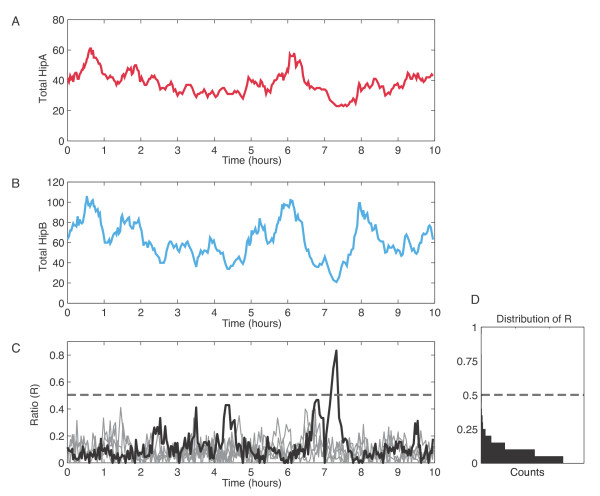
**Noise in wild type HipBA toxin-antitoxin system.(A)** Total HipA concentration in a single simulated cell over time. Note the strong correlation with **(B)**, the total HipB concentration. **(C)** The ratio of free (unbound) HipA to free HipB plus free HipA. The quantity is defined as R. A value of R exceeding 0.5 indicates persistence, shown with a dashed line. Plot shows stochastic data from several simulated cells (gray) and one persistence event (black). The black trace corresponds to the HipA and HipB plots in **(A)** and **(B)**. **(D)** Histogram showing distributions of R.

Figure 2a-b shows the total concentrations of HipA and HipB from one simulation. The two protein levels are correlated due to their cotranscriptional expression. However, they are not perfectly in sync due to stochastic fluctuations in translation and degradation, so the ratio R fluctuates over time (Figure 2c). This phenotypic variation is the source of persistence in the model; individual cells can enter the persistence state due to natural variability in gene expression. Persistence is a rare event and most of the variability in expression levels is under the threshold required to produce a persister. This fact is underscored by Figure 2d, which shows the distribution of R values for the system.

**Figure 3:**
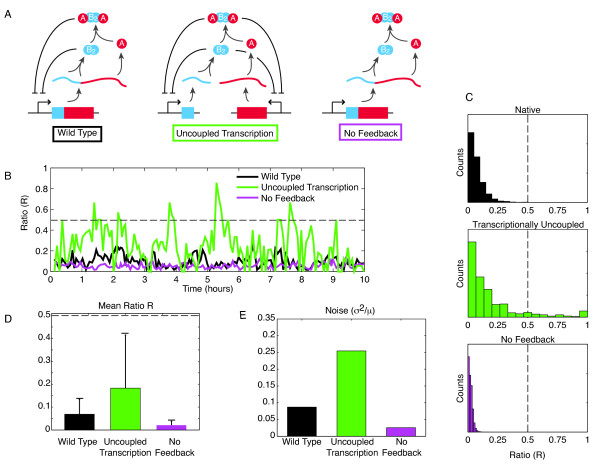
**Alternative regulatory circuit architectures.(A)** Wild type, uncoupled transcription, and no feedback network models. **(B)** Sample simulation traces of the HipA and HipB ratio for the alternative circuit topologies. **(C)** Histograms showing distributions of R (ratio) values for the three circuit topologies. **(D)** Mean value of R. Note that with transcriptionally uncoupled genes, the mean ratio is closer to persistence. With no feedback, the ratio is further from the persistence threshold. Error bars show standard deviation. **(E)** Comparison of the noise (variance divided by mean, σ^2^/μ) for the alternative circuit architectures. The transcriptionally uncoupled network shows increased noise; without feedback there is decreased noise. The combination of elevated mean and noise in the uncoupled transcription case increases the likelihood of persistence events, while the opposite is true for the no feedback case.

Next, we considered alternative architectures for the HipBA system with the goal of understanding how the regulatory topology affects noise and what the implications are for persistence. We first considered a case where *hipB* and *hipA* expression are transcriptionally uncoupled. In the natural system, *hipB* and *hipA* are on the same operon and are transcribed together. When transcribed independently, as shown in Figure 3a, the noise in the system increases, as does the mean of R (Figure 3b-e). Both of these factors lead to increased persistence as compared to the native system. We next constructed a model without the negative feedback loops. In the new system, B_2_ and AB_2_A do not repress the promoter as they do in the wild type system. Without feedback, expression of *hipB* and *hipA* is increased, as transcription is no longer repressed. The system is still noisy, but the mean of R and the noise in the system both decrease (Figure 3b-e), so persistence events are less common.

A system with increased persistence would be better suited for conditions where extreme environmental stress occurs frequently or for extended periods of time. Although the population growth rate would be severely compromised, cells would have an increased likelihood of surviving extreme or long-term environmental stresses, such as long-term nutrient deprivation or antibiotic treatment. Conversely, a system with decreased persistence would benefit from increased growth rates and thrive in environments where stresses are few and far between. A previous model of persistence has shown that the optimal frequency of persistence events is closely tied to the frequency of environmental change [37]. The regulatory topology of the HipBA network sets a frequency of entry into persistence, which may be a strong indicator of the frequency with which adverse environments are encountered.

Evolutionarily, it would be possible to achieve either of the alternative circuit topologies discussed here through straightforward mutation or duplication events. Given that stochastic fluctuations in phenotypic states are the likely source of persisters, it is necessary for the regulatory architecture to produce sufficient variability to insure against rare but catastrophic environmental stresses. This suggests that the HipBA toxin-antitoxin system has evolved to allow a specific amount of noise, and thus persistence, to balance between optimal growth and survival against environmental threats.

## Conclusions

We have developed a model of the regulatory interactions that control expression of the *hipBA* operon. The model incorporates recent experimental findings on toxin-antitoxin complex formation [27], includes active degradation of proteins [21,31,32], and repression of *hipBA* gene expression by both the HipB dimer and the toxin-antitoxin complex [27]. Using a deterministic model based on the biochemical reactions we find that the system exhibits monostable behavior for biologically realistic parameters. Thus, stochastic fluctuations in expression of HipB and HipA are likely responsible for the spontaneous formation of persisters. A stochastic model of the biochemical system demonstrates that persister formation is possible when R, the ratio of free HipA to free HipA plus free HipB exceeds a threshold. By comparing two alternative gene circuit architectures we demonstrate that the level of noise, and thus the frequency of persistence, is highly dependent on the regulatory topology. Through mutations it would be possible to achieve systems with either higher or lower noise than the wild type system. Populations must balance the tradeoff between frequency of persistence and growth. We conclude that straightforward changes to the regulatory architecture and associated parameters influence noise levels, which define the frequency of persistence, suggesting that the wild type persistence frequency can be the subject of evolutionary tuning.

## Competing interests

The authors declare that they have no competing interests.

## Additional File

## Authors’ contributions

RSK and MJD designed the research. RSK developed the model, performed the simulations, and analyzed the data. RSK and MJD wrote the manuscript. Both authors read and approved the final manuscript.

## Supplementary Material

Additional file 1 Supplementary material file. Includes reduced order model, additional simulations, and additional modeling details [38]. Click here for file

## References

[B1] Alon U An Introduction to Systems Biology: Design Principles of Biological Circuits 2007

[B2] Cağatay T Architecture-dependent noise discriminates functionally analogous differentiation circuits Cell 2009 139 3 512 522 10.1016/j.cell.2009.07.046 19853288

[B3] Kittisopikul M Suel GM Biological role of noise encoded in a genetic network motif Proc Natl Acad Sci U S A 2010 107 30 13300 13305 10.1073/pnas.1003975107 20616054 PMC2922135

[B4] Austin DW Gene network shaping of inherent noise spectra Nature 2006 439 7076 608 611 10.1038/nature04194 16452980

[B5] Becskei A Serrano L Engineering stability in gene networks by autoregulation Nature 2000 405 6786 590 593 10.1038/35014651 10850721

[B6] Savageau M Comparison of classical and autogenous systems of regulation in inducible operons Nature 1974 252 546 549 10.1038/252546a0 4431516

[B7] Zhang Z Qian W Zhang J Positive selection for elevated gene expression noise in yeast Mol Syst Biol 2009 5 299 19690568 10.1038/msb.2009.58 PMC2736655

[B8] Bar-Even A Noise in protein expression scales with natural protein abundance Nat Genet 2006 38 6 636 643 10.1038/ng1807 16715097

[B9] Newman JRS Single-cell proteomic analysis of S. cerevisiae reveals the architecture of biological noise Nature 2006 441 7095 840 846 10.1038/nature04785 16699522

[B10] Fraser H Noise minimization in eukaryotic gene expression PLoS Biol 2004 2 6 834 838 10.1371/journal.pbio.0020137 PMC400249 15124029

[B11] Dublanche Y Noise in transcription negative feedback loops: simulation and experimental analysis Mol Syst Biol 2006 2 41 16883354 10.1038/msb4100081 PMC1681513

[B12] Nevozhay D Negative autoregulation linearizes the dose–response and suppresses the heterogeneity of gene expression Proc Natl Acad Sci U S A 2009 106 13 5123 5128 10.1073/pnas.0809901106 19279212 PMC2654390

[B13] Simpson ML Cox CD Sayler GS Frequency domain analysis of noise in autoregulated gene circuits Proc Natl Acad Sci U S A 2003 100 8 4551 4556 10.1073/pnas.0736140100 12671069 PMC404696

[B14] Thattai M Shraiman BI Metabolic switching in the sugar phosphotransferase system of Escherichia coli Biophys J 2003 85 2 744 754 10.1016/S0006-3495(03)74517-2 12885625 PMC1303199

[B15] Wolf DM Vazirani VV Arkin AP Diversity in times of adversity: probabilistic strategies in microbial survival games J Theor Biol 2005 234 2 227 253 10.1016/j.jtbi.2004.11.020 15757681

[B16] Brock A Chang H Huang S Non-genetic heterogeneity–a mutation-independent driving force for the somatic evolution of tumours Nat Rev Genet 2009 10 5 336 342 10.1038/nrg2556 19337290

[B17] Charlebois DA Abdennur N Kaern M Gene expression noise facilitates adaptation and drug resistance independently of mutation Phys Rev Lett 2011 107 21 218101 22181928 10.1103/PhysRevLett.107.218101

[B18] Fraser D Kaern M A chance at survival: gene expression noise and phenotypic diversification strategies Mol Microbiol 2009 71 6 1333 1340 10.1111/j.1365-2958.2009.06605.x 19220745

[B19] Zhuravel D Phenotypic impact of regulatory noise in cellular stress-response pathways Syst Synth Biol 2010 4 2 105 116 10.1007/s11693-010-9055-2 20805931 PMC2923296

[B20] Lewis K Persister cells, dormancy and infectious disease Nat Rev Microbiol 2007 5 1 48 56 10.1038/nrmicro1557 17143318

[B21] Gerdes K Christensen S Lobner-Olesen A Prokaryotic toxin-antitoxin stress response loci Nat Rev Microbiol 2005 3 5 371 382 10.1038/nrmicro1147 15864262

[B22] Lewis K Persister cells Annu Rev Microbiol 2010 64 357 372 10.1146/annurev.micro.112408.134306 20528688

[B23] Bigger J Treatment of staphylococcal infections with penicillin by intermittent sterilisation Lancet 1944 ii 497 500

[B24] Christensen S Gerdes K RelE toxins from Bacteria and Archaea cleave mRNAs on translating ribosomes, which are rescued by tmRNA Mol Microbiol 2003 48 5 1389 1400 10.1046/j.1365-2958.2003.03512.x 12787364

[B25] Lou C Li Z Ouyang Q A molecular model for persister in E. coli J Theor Biol 2008 255 2 205 209 10.1016/j.jtbi.2008.07.035 18721814

[B26] Ramage HR Connolly LE Cox JS Comprehensive Functional Analysis of Mycobacterium tuberculosis Toxin-Antitoxin Systems: Implications for Pathogenesis, Stress Responses, and Evolution PLoS Genet 2009 5 12 e1000767 10.1371/journal.pgen.1000767 20011113 PMC2781298

[B27] Schumacher MA Molecular Mechanisms of HipA-Mediated Multidrug Tolerance and Its Neutralization by HipB Science 2009 323 5912 396 401 10.1126/science.1163806 19150849 PMC2764309

[B28] Balaban N Bacterial persistence as a phenotypic switch Science 2004 305 5690 1622 1625 10.1126/science.1099390 15308767

[B29] Black D Irwin B Moyed H Autoregulation of Hip, an operon that affects lethality due to inhibition of peptidoglycan of DNA-synthesis J Bacteriol 1994 176 13 4081 4091 8021189 10.1128/jb.176.13.4081-4091.1994 PMC205607

[B30] Vilar JMG Mechanisms of noise-resistance in genetic oscillators Proc Natl Acad Sci U S A 2002 99 9 5988 5992 10.1073/pnas.092133899 11972055 PMC122889

[B31] Rotem E Regulation of phenotypic variability by a threshold-based mechanism underlies bacterial persistence Proc Natl Acad Sci U S A 2010 107 28 12541 12546 10.1073/pnas.1004333107 20616060 PMC2906590

[B32] Bernstein J Global analysis of Escherichia coli RNA degradosome function using DNA microarrays Proc Natl Acad Sci U S A 2004 101 9 2758 2763 10.1073/pnas.0308747101 14981237 PMC365694

[B33] Gillespie DT Exact Stochastic Simulation Of Coupled Chemical-Reactions J Phys Chem 1977 81 25 2340 2361 10.1021/j100540a008

[B34] Acar M Becskei A van Oudenaarden A Enhancement of cellular memory by reducing stochastic transitions Nature 2005 435 7039 228 232 10.1038/nature03524 15889097

[B35] Choi PJ A stochastic single-molecule event triggers phenotype switching of a bacterial cell Science 2008 322 5900 442 446 10.1126/science.1161427 18927393 PMC2819113

[B36] Dhar N McKinney JD Microbial phenotypic heterogeneity and antibiotic tolerance Curr Opin Microbiol 2007 10 1 30 38 10.1016/j.mib.2006.12.007 17215163

[B37] Kussell E Bacterial persistence: A model of survival in changing environments Genetics 2005 169 4 1807 1814 10.1534/genetics.104.035352 15687275 PMC1449587

[B38] Murray JD Mathematical Biology I 2002 Springer,

