# Supplementary material for: Modeling suggests that gene circuit architecture controls phenotypic variability in a bacterial persistence network
Source: BMC Syst Biol. 2012 May 20;6:47. doi: 10.1186/1752-0509-6-47 (PMC3434061; doi:10.1186/1752-0509-6-47)
Supplement: Additional file 1 — Supplementary material file. Includes reduced order model, additional simulations, and additional modeling details [38]. [file 1752-0509-6-47-S1.pdf]

1 Reduced Order Model

The differential equations given in Eq. 1 of the main text describe the full system dynamics. To gain further insight into the possible dynamics and test for bistability, we reduced the model to two state variables by making quasi-steady-state assumptions [1]. Specifically, we set the rate of change of the following differential equations to zero: $\frac{d[P]}{dt}$, $\frac{d[P']}{dt}$, $\frac{d[P'']}{dt}$, $\frac{d[M]}{dt}$, $\frac{d[B]}{dt}$, and $\frac{d[B_2]}{dt}$, focusing on the slow dynamics of $[A]$ and $[AB_2A]$. Thus, it is possible to simplify the system to two differential equations, given by

$$\frac{d[A]}{dt} = \beta_A[M] - \mu[A]^2[B_2] + \mu_R[AB_2A] - \delta_A[A] \quad (1)$$

$$\frac{d[AB_2A]}{dt} = \frac{1}{2}\mu[A]^2[B_2] - \frac{1}{2}\mu_R[AB_2A] - \theta_{AB_2A}[P][AB_2A] + \gamma_{AB_2A}[P''] - \delta_{AB_2A}[AB_2A], \quad (2)$$

where

$$[P] = T(1-\lambda)\left(1 - \frac{\rho[B_2]}{\gamma_{B_2} + \rho[B_2]}\right) \quad (3)$$

$$[P'] = \frac{\rho T[B_2]}{\gamma_{B_2} + \rho[B_2]} \quad (4)$$

$$[P''] = \lambda T\left(1 - \frac{\rho[B_2]}{\gamma_{B_2} + \rho[B_2]}\right) \quad (5)$$

$$[M] = \frac{\sigma\rho T[B_2]}{\gamma_{B_2} + \rho[B_2]} + \beta \quad (6)$$

$$[B] = -\frac{\delta_B}{2\beta_{B_2}} + \frac{\sqrt{\delta_B^2 + 4\beta_B\beta_{B_2}\left(\frac{\sigma\rho T[B_2]}{\gamma_{B_2}+\rho[B_2]} + \beta\right)}}{2\beta_{B_2}}. \quad (7)$$

$T = [P] + [P'] + [P'']$, which is a constant since the promoter switches between these three states, but is never created or destroyed. Other variables are given by

$$\lambda = \frac{\theta_{AB_2A}[AB_2A]}{\gamma_{AB_2A} + \theta_{AB_2A}[AB_2A]}$$

$$\rho = \theta_{B_2}(1-\lambda)$$

$$\sigma = \frac{-\alpha(1-\lambda) + \alpha_{\beta_2} - \alpha_{AB_2A}\lambda}{\delta_M}$$

$$\beta = \frac{\alpha T(1-\lambda) + \alpha_{AB_2A}\lambda T}{\delta_M}.$$

The equation for $[B_2]$ cannot be solved analytically, however it can be found with numerical root finding methods. Specifically, the following equation is solved for $[B_2]$, applying Eqs. 3–7, which are functions of $[B_2]$ and the known reduced order model state variables:

$$\frac{1}{2}\beta_{B_2}[B]^2 - \frac{1}{2}\mu[A]^2[B_2] + \frac{1}{2}\mu_R[AB_2A] - \theta_{B_2}[P][B_2] + \gamma_{B_2}[P'] - \delta_{B_2}[B_2] = 0. \quad (8)$$

Fig. 1 shows that the reduced order model is an excellent approximation of the full system after the fast transients have died out. The system response during the initial transients is not fully captured by the reduced order model, which is a limitation of this approach. However, the long-time behavior of the reduced order model shows good agreement with the full system and is used to approximate the full system to test for the existence of multistability.

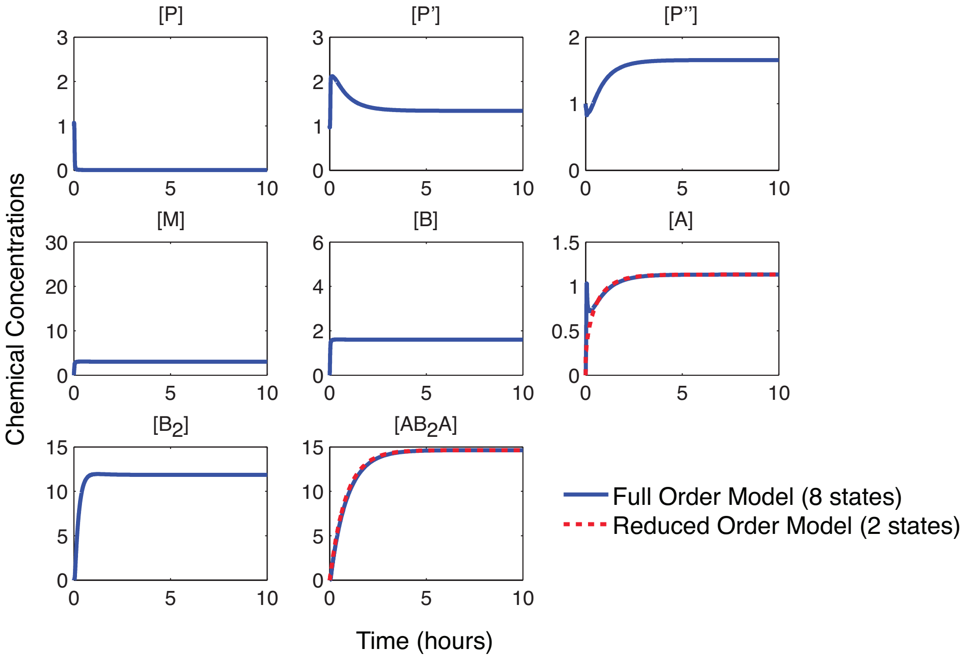

Figure 1: Reduced order model compared to the full model

2 Additional Simulations

The system was simulated with the full set of differential equations for different initial conditions. Fig. 2 shows representative solutions, all of which converge to a single equilibrium point. Ten simulations of HipB, HipA, the HipB dimer, and the HipB-HipA complex are shown. HipB and HipA initial conditions were varied starting at zero and increased to ten in increments of one. The HipB dimer and HipBA complex were varied starting at zero and increased to fifty in increments of five.

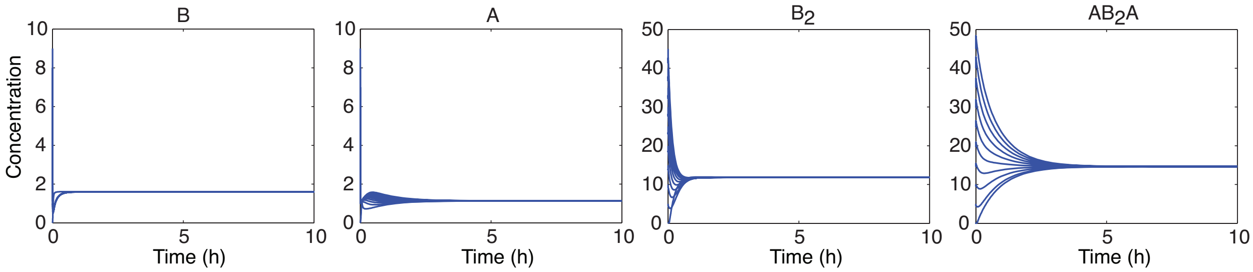

Figure 2: Examples of deterministic model solutions

3 Alternative Models

3.1 Uncoupled Transcription

The following biochemical reactions model the uncoupled transcription system, where *hipB* and *hipA* are on separate operons, but have the same regulation.

Reaction	Description
$P_B \xrightarrow{\alpha} P_B + M_B$<br>$P'_B \xrightarrow{\alpha_{B_2}} P'_B + M_B$<br>$P''_B \xrightarrow{\alpha_{AB_2A}} P''_B + M_B$	Transcription of *hipB* from different promoter states
$P_A \xrightarrow{\alpha} P_A + M_A$<br>$P'_A \xrightarrow{\alpha_{B_2}} P'_A + M_A$<br>$P''_A \xrightarrow{\alpha_{AB_2A}} P''_A + M_A$	Transcription of *hipA* from different promoter states
$P_B + B_2 \underset{\gamma_{B_2}}{\overset{\theta_{B_2}}{\rightleftharpoons}} P'_B$<br>$P_B + AB_2A \underset{\gamma_{AB_2A}}{\overset{\theta_{AB_2A}}{\rightleftharpoons}} P''_B$<br>$P_A + B_2 \underset{\gamma_{B_2}}{\overset{\theta_{B_2}}{\rightleftharpoons}} P'_A$<br>$P_A + AB_2A \underset{\gamma_{AB_2A}}{\overset{\theta_{AB_2A}}{\rightleftharpoons}} P''_A$	Transcription factor / promoter binding
$M_B \xrightarrow{\delta_M} \emptyset$<br>$M_A \xrightarrow{\delta_M} \emptyset$	mRNA degradation
$M_B \xrightarrow{\beta_B} M_B + B$<br>$M_A \xrightarrow{\beta_A} M_A + A$	Translation
$B + B \xrightarrow{\beta_{B_2}} B_2$<br>$2A + B_2 \underset{\mu_R}{\overset{\mu}{\rightleftharpoons}} AB_2A$	Protein complex formation
$B \xrightarrow{\delta_B} \emptyset$<br>$A \xrightarrow{\delta_A} \emptyset$<br>$B_2 \xrightarrow{\delta_{B_2}} \emptyset$<br>$AB_2A \xrightarrow{\delta_{AB_2A}} \emptyset$	Protein degradation

3.2 No Feedback

The biochemical reactions describing the system with no feedback are given below.

Reaction	Description
$P \xrightarrow{\alpha} P + M$	Transcription
$M \xrightarrow{\beta_B} M + B$<br>$M \xrightarrow{\beta_A} M + A$	Translation

$B + B \xrightarrow{\beta_{B_2}} B_2$<br>$2A + B_2 \underset{\mu_R}{\overset{\mu}{\rightleftharpoons}} AB_2A$	Protein complex formation
$M \xrightarrow{\delta_M} \emptyset$	mRNA degradation
$B \xrightarrow{\delta_B} \emptyset$<br>$A \xrightarrow{\delta_A} \emptyset$<br>$B_2 \xrightarrow{\delta_{B_2}} \emptyset$<br>$AB_2A \xrightarrow{\delta_{AB_2A}} \emptyset$	Protein degradation

References

[1] J. D. Murray. Mathematical Biology I. Springer 2002.
